# Craniomaxillofacial trauma in immature dogs–etiology, treatments, and outcomes

**DOI:** 10.3389/fvets.2022.932587

**Published:** 2022-08-15

**Authors:** Elias Wolfs, Boaz Arzi, Jose Guerrero Cota, Philip H. Kass, Frank J. M. Verstraete

**Affiliations:** ^1^School of Veterinary Medicine, William R. Pritchard Veterinary Medical Teaching Hospital, University of California, Davis, Davis, CA, United States; ^2^Department of Surgical and Radiological Sciences, School of Veterinary Medicine, University of California, Davis, Davis, CA, United States; ^3^Department of Population Health and Reproduction, School of Veterinary Medicine, University of California, Davis, Davis, CA, United States

**Keywords:** computed tomography, cone beam CT, fracture, temporomandibular joint, skull

## Abstract

Treatment of craniomaxillofacial (CMF) trauma in dogs often requires a multidisciplinary approach and a thorough understanding of the CMF anatomical structures involved. This retrospective study aimed to utilize computed tomography (CT) studies of immature dogs evaluated for CMF trauma and to describe common fracture locations, treatment modalities, and complications, as well as the fracture healing outcomes. The medical records and CT studies of 94 dogs under 1 year of age over a 13-year period were evaluated. The skeletal location of CMF fractures, as well as the severity of displacement and fragmentation of each fracture, was recorded. Case demographic data and trauma etiology were also recorded. Animal bites accounted for the majority of trauma (71.0%). The most likely bone or region to be fractured was the maxillary bones, followed by the molar region of the mandibles. Up to 37 bones or specific regions were fractured in any given patient, with an average of 8.8 ± 3.1 fractured bones or regions per dog. Rostral mandibular trauma was associated with intra-articular fractures of the temporomandibular joint (*p* = 0.016). Patients sustained concomitant injuries in 32% of the cases. Muzzle therapy was the main treatment performed for most dogs (53.2%), followed by soft tissue closure (47.9%) and selective dental extractions (27.6%). Healing complications were recorded in 71.6% of the dogs, with malocclusion being the most reported complication (55.2%), and associated with dentate mandibular jaw fractures (*p* = 0.05). The average number of complications per dog was 2.4. No statistically significant association was found between treatment modality and healing outcome. There was a positive correlation between the severity of fracture fragmentation and displacement and a negative healing outcome (all rho >0.7). Further treatment was required in 55.6% of the dogs. Additional dental extractions were performed in 77.7% of patients. Healing complications were common in the immature CMF trauma case. Thus, the need for a comprehensive assessment of the entire CMF region during the initial visit, as well as follow-up, preferably using CT or cone beam CT, is underscored.

## Introduction

Craniomaxillofacial (CMF) trauma is a relatively common reason for which dogs are presented to veterinarians on an emergency basis. CMF fractures in dogs occur more frequently in small and juvenile dogs (1). In addition, reported treatment modalities, bone healing, and postoperative complications historically did not distinguish between the age of the dog or the stage of development of the teeth or jaws, however with recently reported treatment and outcomes of juvenile mandibular fractures (2–7). The veterinarian should consider multiple factors prior to deciding on the optimal treatment. These include location and type of fracture, involvement of teeth, presence of teeth for the anchorage of interdental wiring and intraoral splinting, bone quality, available methods for repair, and the operator's skills. Stabilization of CMF fractures can be achieved with non-invasive (muzzling) and minimally invasive techniques (maxillomandibular fixation, interdental wiring, and intraoral splinting) or invasive techniques (intraosseous wiring, external skeletal fixation, and bone plating) (5, 8–10). Stabilization techniques in juvenile dogs provide limited treatment options because of the presence of developing tooth buds in the jaw bone, the absence of sufficiently erupted permanent teeth or exfoliating deciduous teeth, immature bone, and the potential for interference with dental and jaw development (6, 8, 9, 11–13). Complications derived from CMF fractures include malunion or non-union, malocclusion, infection, and periodontal and/or endodontic disease of teeth in or near the jaw fracture line (4, 6, 13, 14). Dental and jaw developmental abnormalities are especially of concern in immature dogs that are still growing (7, 9, 13, 14).

According to Scott and Fuller (15), the juvenile period in dogs runs from ~12 weeks (the postulated end of the socialization period) until 6 months of age or later, corresponding to the onset of sexual maturation (i.e., puberty). The canine adolescence period, marked by changes in circulating gonadal hormones, is reported to be between ~6 months and 1–2 years of age depending on the individual and breed (16). The authors of this article use the term immature to include juvenile and adolescent dogs.

The human medical literature reports that a minimalist approach was historically taken in the management of pediatric facial fractures because of concern for interference with the growth and development of the pediatric facial skeleton. Closed reduction and maxillomandibular fixation (MMF) were initially the treatment methods of choice for all displaced facial fractures (17, 18). Now, open reduction and internal fixation (ORIF) is the gold standard treatment for displaced fractures; and the benefits of ORIF are quite apparent, namely fixation in three dimensions, the potential for no or less time spent in MMF, decreasing airway risk, and improving nutrition and tolerance (19). However, disruption of the periosteum and vascular supply as well as the creation of scars may interfere with the future growth of the affected area (19).

Given the anatomically complex and overlapping nature of structures in the CMF region, the diagnostic yield of CT in identifying fractures is greater than that of skull radiographs (20, 21). Therefore, CT is considered the gold standard for craniofacial imaging in people and in veterinary species, and there is increasing recognition that three-dimensional and multiplanar reconstructions are essential for accurate diagnosis and optimal treatment planning (22, 23). While utilizing the two-dimensional aspects of CT is essential for the smaller or more deeply located fractured structures in the CMF region, it is well-recognized that the two-dimensional and three-dimensional modalities are best utilized together (23–25). In some situations, CT is also being utilized intraoperatively and has been shown to change clinical decision-making (26). Fortunately, access to CT in veterinary practice is on a trajectory that may improve the accuracy of diagnosis in CMF trauma cases.

There is a paucity of data with respect to the management of CMF fractures in immature dogs utilizing CT, as well as treatment methods, complications, and fracture healing outcomes. The primary objectives of this retrospective study were: (1) to describe common fracture locations, treatment modalities, and complications and (2) the fracture healing outcomes. We hypothesized that the immature dog is at increased risk of developing healing complications influenced by fracture type and location as well as the treatment modality. In addition, we hypothesized that young dogs with their good innate bone healing capacity will result in bone healing despite the severity of displacement or fragmentation, provided that appropriate treatment is performed.

## Materials and methods

### Case selection

The electronic medical record database of the UC Davis Veterinary Medical Teaching Hospital was searched for immature dogs that had been presented for evaluation and treatment following CMF trauma between the years 2008 and 2020. For inclusion, all dogs under 1 year of age must have undergone CT [conventional CT and/or cone-beam CT (CBCT)] at the initial visit. Exclusion criteria were as follows: trauma that had occurred >1 week prior to presentation, imaging performed with CT slice thickness of > 1.3 mm, patients that were older than 1 year of age, and those for whom either the medical record or CT study was incomplete (e.g., the caudal most portion of the skull had not been included in the images). Cases were excluded if the trauma occurred > 7 days prior to presentation due to the following concerns: (1) early signs of fracture repair and bone remodeling may make fracture identification more difficult and (2) further displacement may have occurred since the trauma. Exclusion of cases if the slice thickness was > 1.3 mm was chosen as a compromise between maximizing the number of cases that were included in the study while simultaneously ensuring that slice thickness was not so large that small or incomplete fractures could be missed.

### Image acquisition and evaluation

All dogs underwent conventional CT (HiSpeed FX/i or LightSpeed16, GE Healthcare, Waukesha, WI) and/or CBCT (NewTom 5G CBCT Scanner, NewTom, Verona, Italy) imaging at their initial visit. Conventional CT allowed the study to capture those patients in which superior soft tissue imaging was medically necessary (e.g., those with concern for intracranial hemorrhage), those too large for the CBCT field of view, and those who received treatment prior to the availability of CBCT. All DICOM files from each study were viewed using specialized software (InVivo5, Anatomage, San Jose, CA) as previously described (1, 27). Each case was viewed dynamically on medical flat-grade monitors (ASUS PB278Q 27-inch, ASUSTeK Computer Inc., Taipei, Taiwan), allowing the observers to utilize all viewing modes and tools to best assess all fractures. Two observers (JC, EW) viewed all studies and recorded all data after a period of calibration with two board-certified veterinary dentists and oral surgeons (FJMV, BA).

### Fracture evaluation

Each skull was divided into specific bones and regions based on a previous study by our group (1). The region was defined by a relatively large bone divided into smaller entities (e.g., the molar region of the mandible). For each bone and region, it was determined whether each bone or region was fractured. If so, fracture morphology was described in terms of displacement and fragmentation. The degrees of displacement and fragmentation were modeled after the AOCMF fracture classification system in humans (28). For both displacement and fragmentation, a score of 0 indicated no fracture. When scoring displacement, a score of 1 indicated no displacement, a score of 2 minimal displacements with >50% overlap remaining between fragments, and a score of 3 severe displacements with <50% overlap remaining. When scoring fragmentation, a score of 1 indicated an incomplete fracture, a score of 2 a complete fracture, and a score of 3 a comminuted fracture. The fracture was determined to be unilateral or bilateral in nature. If the fracture was bilateral, the highest allocated ordinal value for fragmentation and displacement was used for statistical analysis. Although the use of the term “comminuted” is discouraged by the most recent recommendations in human CMF literature (29), the term and its associated meaning are still pervasive in veterinary medicine and were therefore utilized in this study. A comminuted fracture was defined as a fracture having three or more bone fragments, although “minute” fragments were ignored unless the entire bone or region had been reduced to microfragments (30).

Because the bones that form the temporomandibular joint (TMJ) may be fractured without a fracture extending into the articular space, fractures of the TMJ were recorded separately from fractures of the condylar process, the retroarticular process, and the mandibular fossa of the temporal bone. It was expected that there would be frequent overlap between these fractures. However, recording the instances of a fracture involving the articular surface itself was considered important enough to be coded separately. Similarly, although the cribriform plate is part of the ethmoid bone (31), the possible prognostic implications of having breached the braincase were deemed important enough to record instances of cribriform fracture separately from other ethmoid fractures.

If a fracture occurred along a suture or at a border between two regions, the bone or region on both sides was considered fractured, and the morphology of the fracture was considered separately for each bone or region. All fractures along a suture were considered complete. However, the degree of displacement was recorded individually for the bone on either side of a suture.

For the mandibular symphysis, symphyseal separation of a fibrocartilaginous joint (synchondrosis) was considered to be bilateral. However, if the two sides were unequally displaced, the coding reflected this.

### Fracture etiology and concomitant trauma

For each case, one of the seven different fracture etiologies was assigned including crush or slow velocity trauma, vehicular injury, animal bite, fall from height, ballistic, blunt force, or unknown trauma. Furthermore, dogs were evaluated for additional trauma, such as spinal, cerebral, abdominal, thorax, extremities, ophthalmological, or no known additional trauma.

### Demographic data

Sex (male and female, intact or neutered) and age (in months or a fraction thereof) were recorded. Breed and skull shape were evaluated and grouped into brachycephalic, mesocephalic, or dolichocephalic conformations based on skull indices or reported breed (31, 32). Additionally, the dogs' dentition status was categorized to be either deciduous, mixed, or permanent.

### Treatment methods

**Figure 1:**
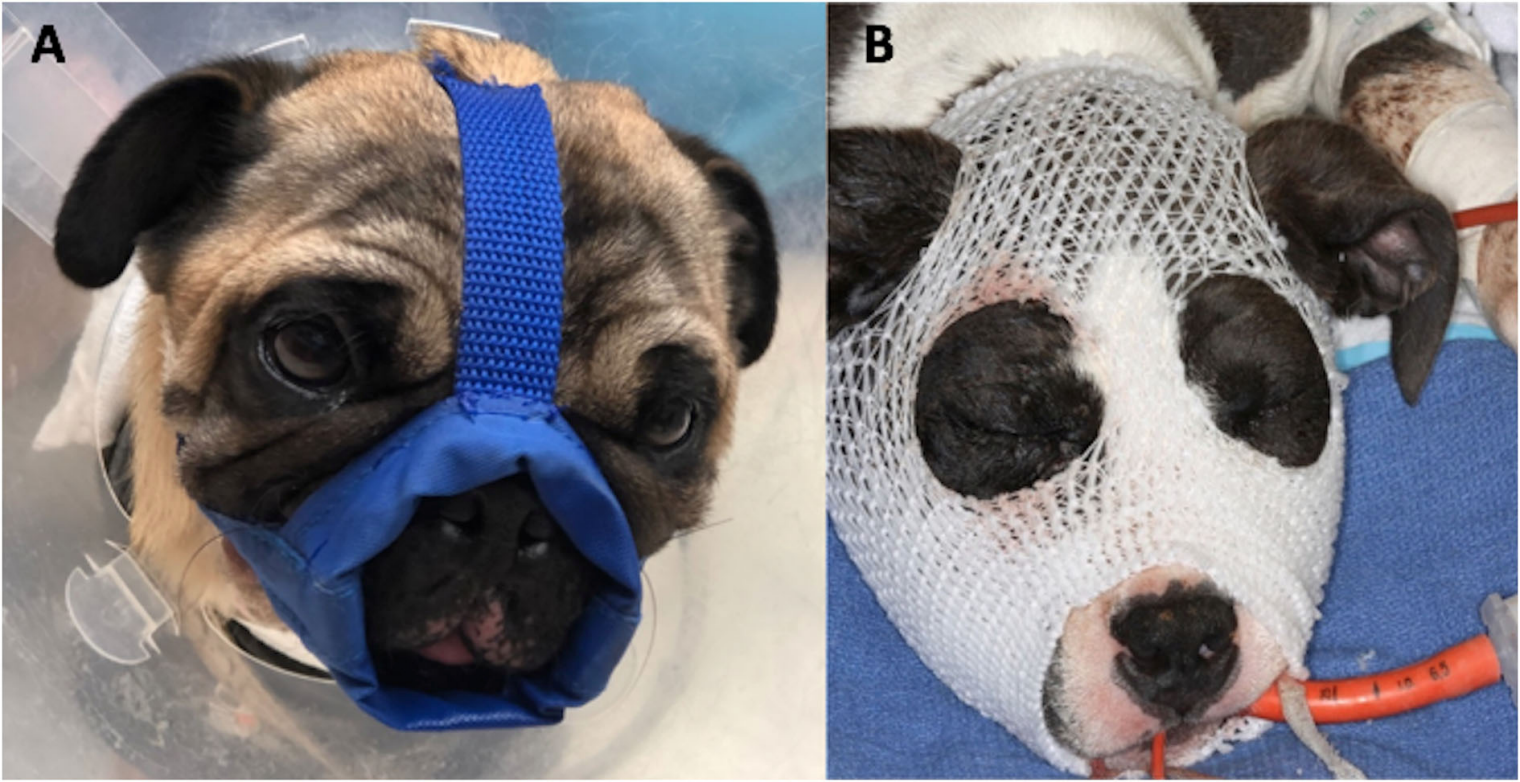
Placement of nylon muzzle holds the jaw in relative normocclusion by decreased mouth opening. It is especially useful for caudal mandibular and temporomandibular (TMJ) fractures and can be custom-made to adapt brachycephalic skull conformation **(A)**. The “face mask” **(B)**, using an elastic dressing retainer, can be applied in cases where maxillary fractures are present as it provides even pressure and stability to the entire face.

**Figure 2:**
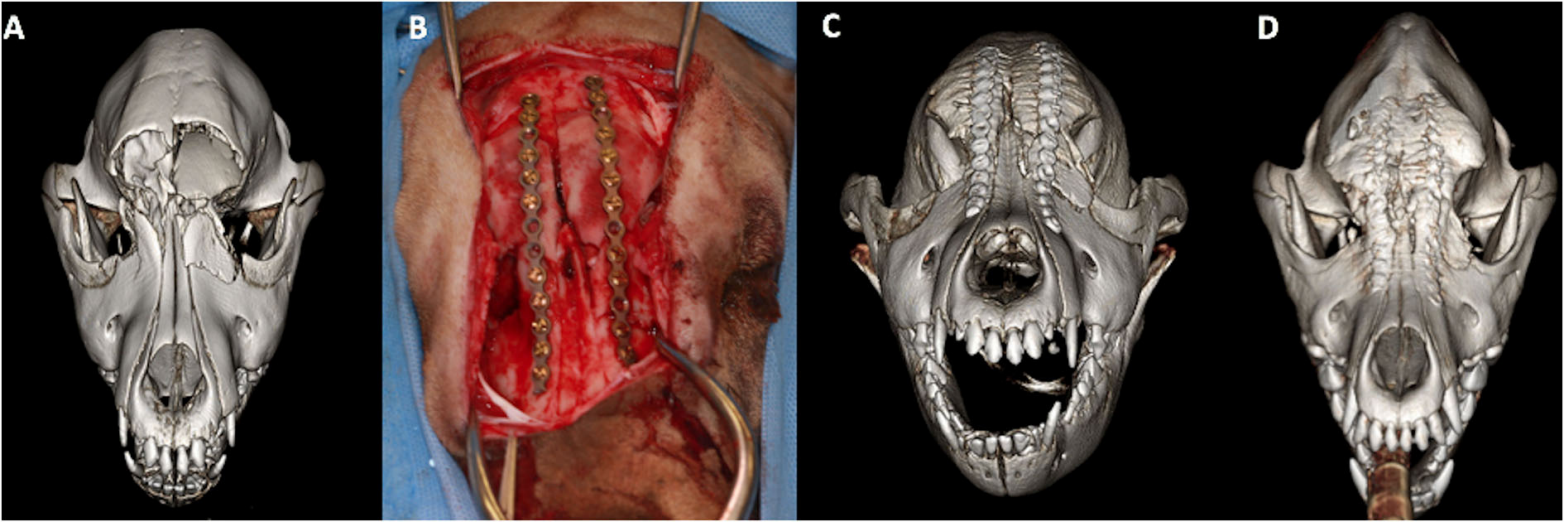
3D-rendered CBCT images **(A,C,D)** and intraoperative image **(B)** of the skull of a 4-month-old golden retriever dog. The preoperative scan **(A)** demonstrates complete and severe displaced fractures of the frontal bones, complete and mildly displaced fracture of the maxillary and lacrimal bones. Open reduction and internal fixation **(B)** using 2.0-mm non-locking titanium plates and screws was performed. The postoperative scan **(C)** demonstrates adequate reduction and apposition of the fracture fragments. The 6-month postoperative CBCT scan **(D)** reveals excellent bone healing without the interference of growth and development of the pediatric facial skeleton.

Treatment methods were assigned for each case. Muzzle therapy was by definition considered conservative treatment management where the use of a tape or nylon muzzle stabilized the fracture fragments (Figure 1). Intraosseous wiring or plating procedure was considered an ORIF technique, or invasive repair technique (Figure 2). Other fracture fragment stabilization techniques included MMF, interdental wire, and composite splint (minimally invasive repair technique). Dogs that underwent a salvage procedure included cases that had a total or considerable bone segment removal with or without dentition (e.g., zygomectomy or rostral mandibulectomy). On the contrary, if the bone segment removal was minimal so that it would not cause any change to the dog's appearance or function, it was categorized as a bone fragment debridement. Soft tissue closure was allocated to the cases where an intra- and/or extraoral laceration repair was performed. Lastly, for each case, we recorded if dental extractions were performed or not included in a salvage procedure. Extractions were performed for the cases where the tooth had a complicated crown fracture, was involved in the fracture line or had stage 3 mobility. Although a combination of treatment methods was possible, separate treatment groups for each possible combination were for statistical analysis not recorded.

### Healing evaluation

When dogs had a re-examination performed at a later date, the medical records were assessed for evaluation of complications. Dehiscence was scored for dogs that were presented with an opening up of a previously repaired surgical site. Wounds and fracture sites that exhibited instability and healing that took longer than 6 weeks were considered delayed healing. Infection was allocated to the cases that had evidence of purulent discharge on the follow-up examination or a positive culture and sensitivity test. A piece of devitalized bone that had become separated from sound bone was classified as a bone sequestrum. If enamel hypoplasia and/or odontodysplasia were noticeable on the follow-up visits, these were categorized under tooth structure defects. For the cases where intrinsically discolored teeth or imaging revealed the failure of the pulp cavity to narrow, were treated as having non-vital teeth. Radiographic or CT evidence of teeth that had failed to erupt was classified as embedded dentition. When a deciduous tooth was still present at the time that a permanent tooth had begun to erupt and was past the normal exfoliation time it was deemed a persistent deciduous tooth. Malpositioned teeth and/or a discrepancy in relative jaw length were assessed as malocclusion. TMJ anomalies were allotted to the cases where CT revealed TMJ ankylosis, pseudo-ankylosis, or early onset of osteoarthritis. Reduced range of motion was reserved for the cases that had restricted jaw opening. Lastly, if neurological deficits or eye-related complications were noticeable, they were lumped in with “other” complications.

**Figure 3:**
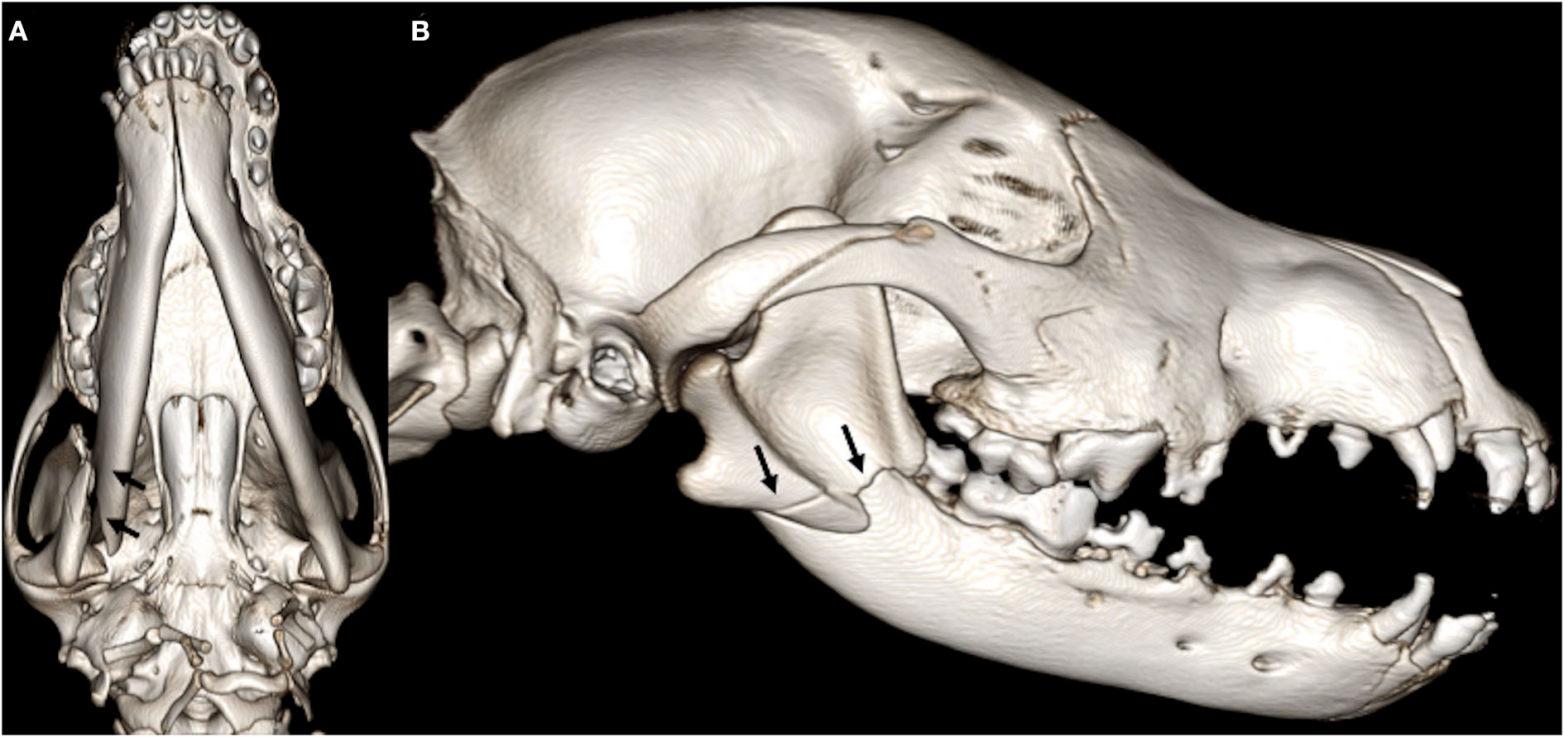
3D-rendered ventral **(A)** and right lateral **(B)** CBCT images of the skull of a 4-month-old Malinois dog. Note the complete, severely displaced fracture involving the right mandibular molar area and the midramus (arrows). Muzzle therapy was elected to stabilize the fracture fragments.

**Figure 4:**
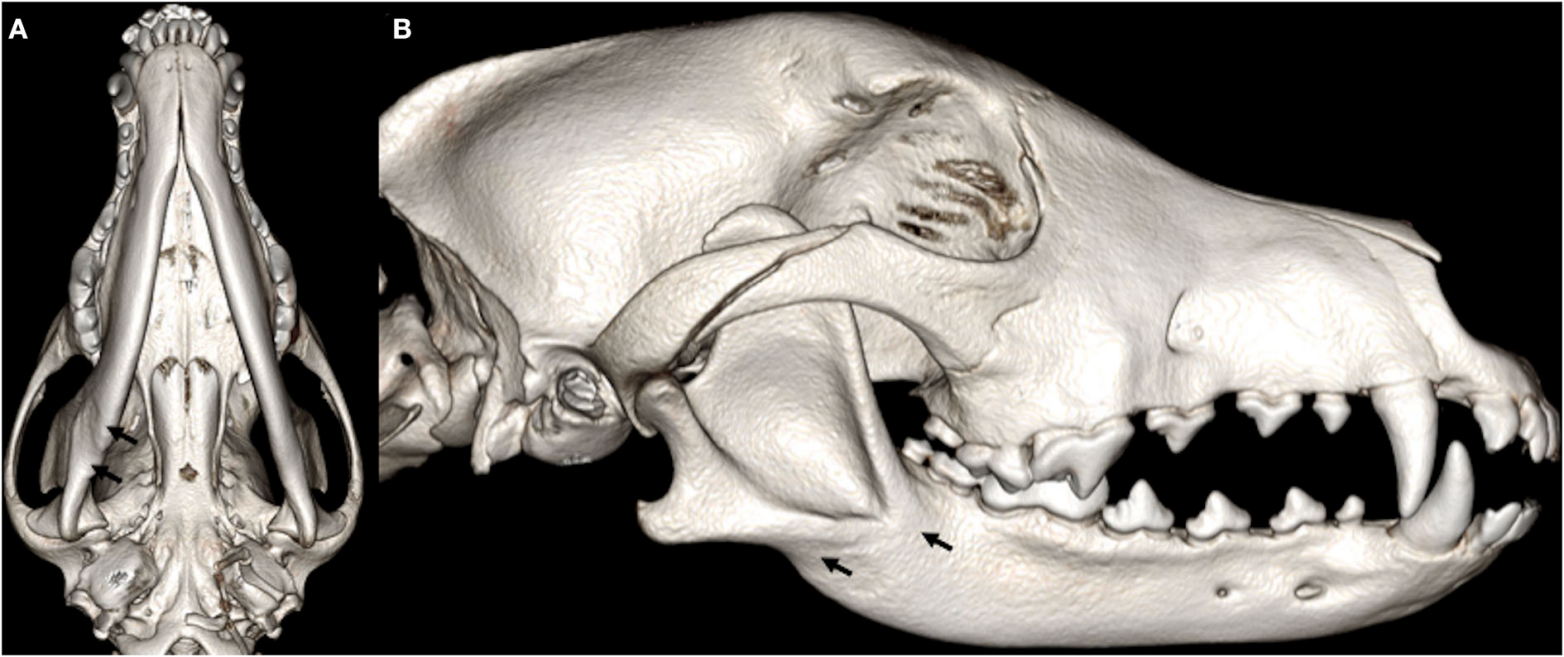
Three-month follow-up: 3D-rendered ventral **(A)** and right lateral **(B)** CBCT images of the skull of the same dog as in Figure 3. The previously described fracture exhibits satisfactory healing with bridging bone formation, callus, and is actively remodeling (arrows).

**Figure 5:**
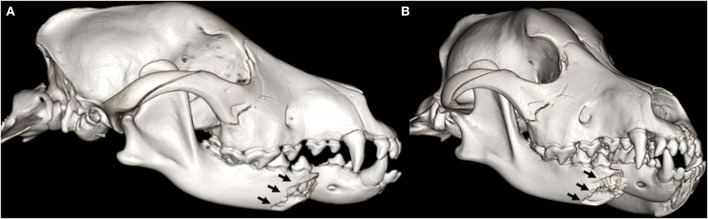
3D-rendered right lateral **(A)** and oblique **(B)** CBCT images of the skull of an 8-month-old Labrador retriever dog. Note the complete fracture with severe displacement of the right premolar area of the mandible (arrows).

**Figure 6:**
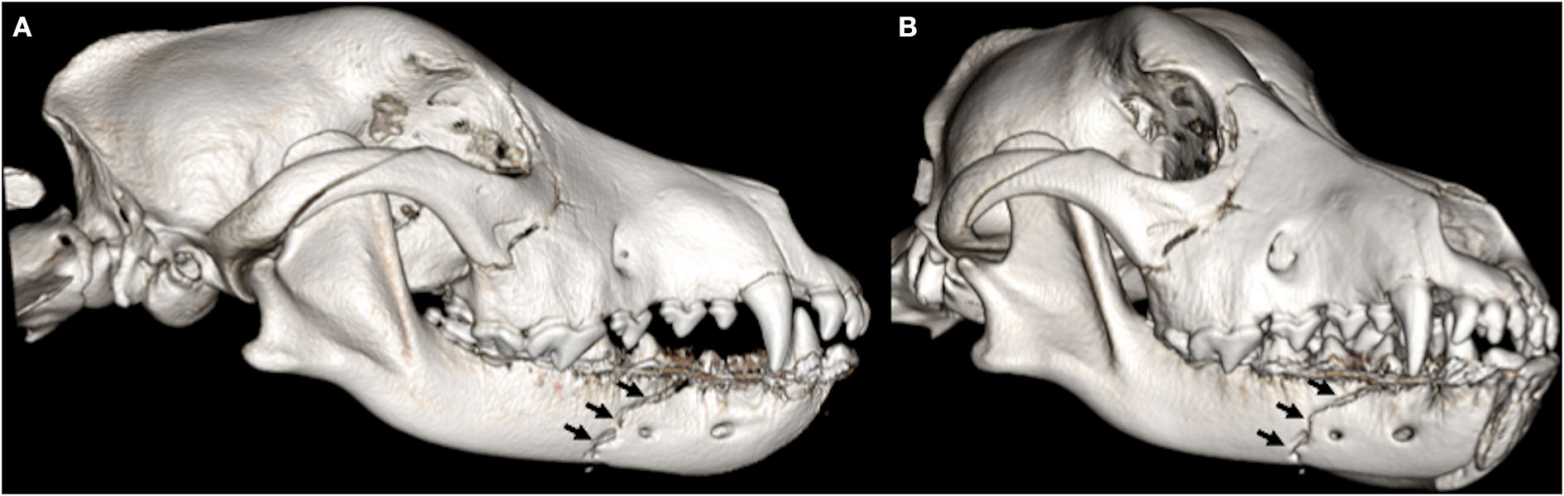
1-month follow-up 3D-rendered right lateral **(A)** and oblique **(B)** CBCT images of the skull of the same dog as in Figure 5. An interdental wire and composite splint were placed to reduce the fracture fragments. There is a persistent fracture gap with no evidence of bridging callus formation suggestive of a non-union.

For the cases that had a follow-up visit by means of a repeat conventional CT or CBCT, the fractures were evaluated for their level of healing. A score of 0 meant that there was no fracture or that the fracture had completely healed in the recheck interim. A score of 1 or 2 indicated satisfactory (Figures 3, 4) or unsatisfactory (Figures 5, 6) healing, respectively. Satisfactory healing was by definition a fracture that showed evidence of bridging callus formation or obliteration of the fracture line and unsatisfactory healing as a lack thereof, malunion, and non-union. If there were less than 10 fractures with follow-up for any given bone or specific bone region, those bones were excluded from statistical analysis due to the imprecision of the statistical estimates.

Lastly, for each follow-up visit, it was determined if further medical intervention was recommended and pursued.

### Statistical methods

The Jonckheere–Terpstra trend test and Spearman correlation coefficients and their 95% confidence intervals calculated using the bootstrap resampling method were used to evaluate the healing outcomes of fractures in relation to the severity of fragmentation and displacement. Fisher's exact test was used to evaluate the association of TMJ fractures with rostral mandibular trauma. For variables associated with the risk of malocclusion, a Kruskal–Wallis and/or Fisher's exact test was conducted. Kruskal–Wallis tests were used to compare healing outcomes between fracture locations and treatment modalities. In addition, for variables significantly associated with healing outcomes, chi-square tests of independence were used to determine which combinations of variable levels and outcome categories contributed to significance. For all analyses, values of *p* ≤ 0.05 were considered significant. All calculations were performed using Stata BE/17.0 statistical software (College Station, TX).

## Results

### Descriptive statistics

**Figure 7:**
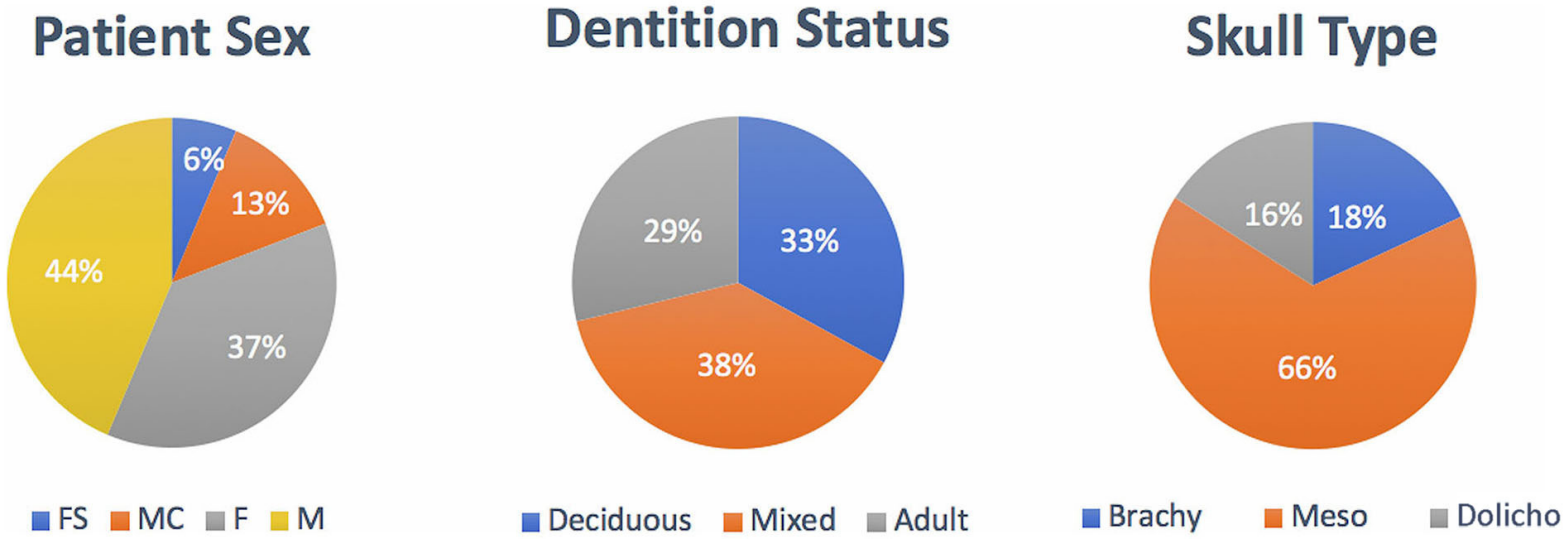
Population distribution by patient sex, patient dentition status, and skull type.

**Figure 8:**
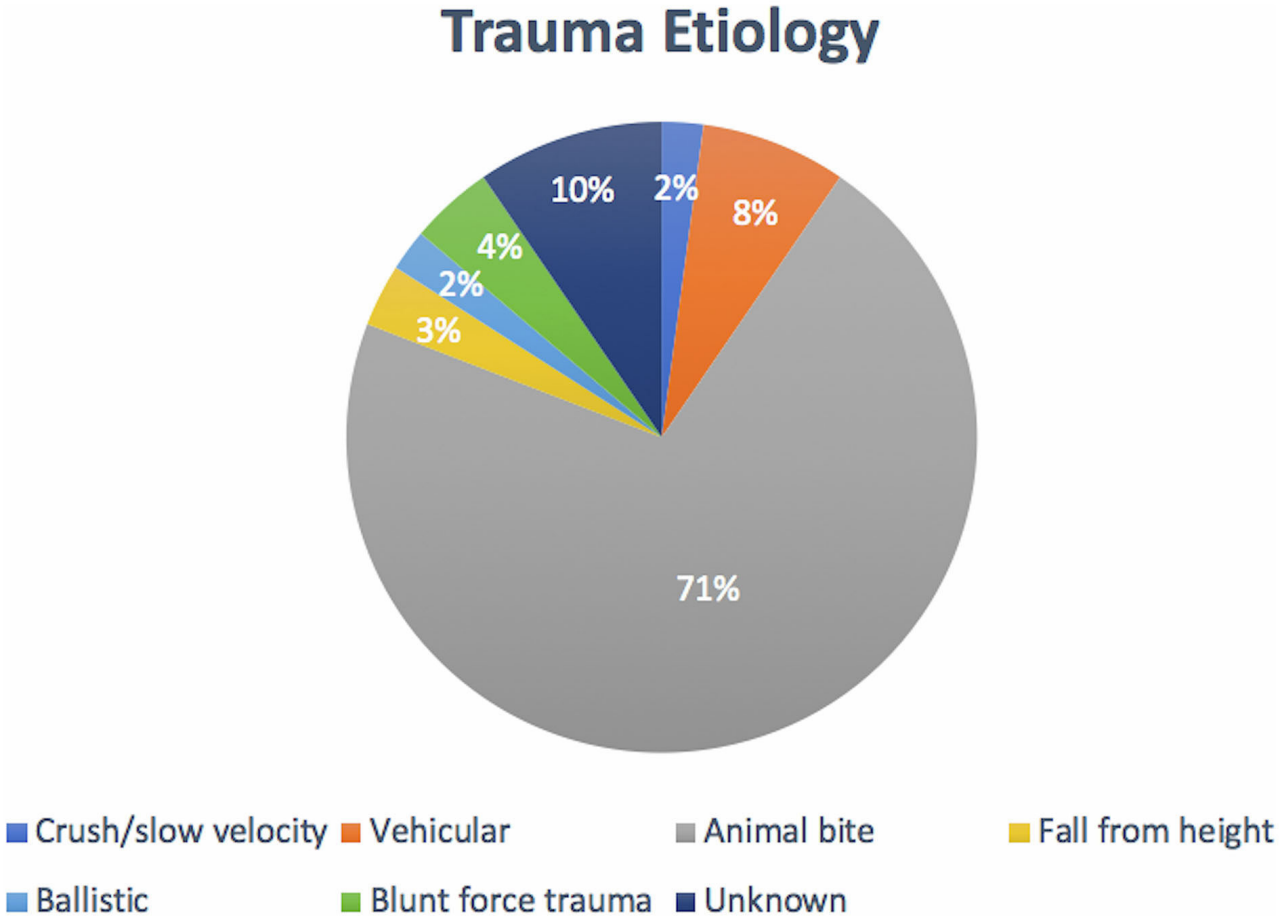
Distribution of trauma etiology. Note that animal bites accounted for the largest proportion of trauma etiologies.

Out of 94 dogs evaluated, 6 were spayed female dogs, 35 were intact female dogs, 12 were neutered male dogs, and 41 were intact male dogs (Figure 7). The ages ranged from 2 days to 12 months, with a mean (SD) age of 4.2 (2.5) months. A relatively equal age distribution between dentition status was noted (Figure 7). The proportion of dogs in each skull conformation was as follows: brachycephalic: 18.0% (*n* = 17), mesocephalic: 66.0% (*n* = 62), and dolichocephalic: 16.0% (*n* = 15) (Figure 7). Incidence of trauma etiology, depicted in Figure 8, demonstrated that animal bites (71.2%) caused the majority of injuries (*n* = 67).

The average number (SD) of fractured regions or bones was 8.8 (3.1) per dog, with up to 37 fractured regions, and only 12.7% of cases (*n* = 12) had a solitary fractured region or bone. Around 64.9% (*n* = 61) of cases had bilateral fractures for at least one bone or region

### Commonly fractured locations

**Figure 9:**
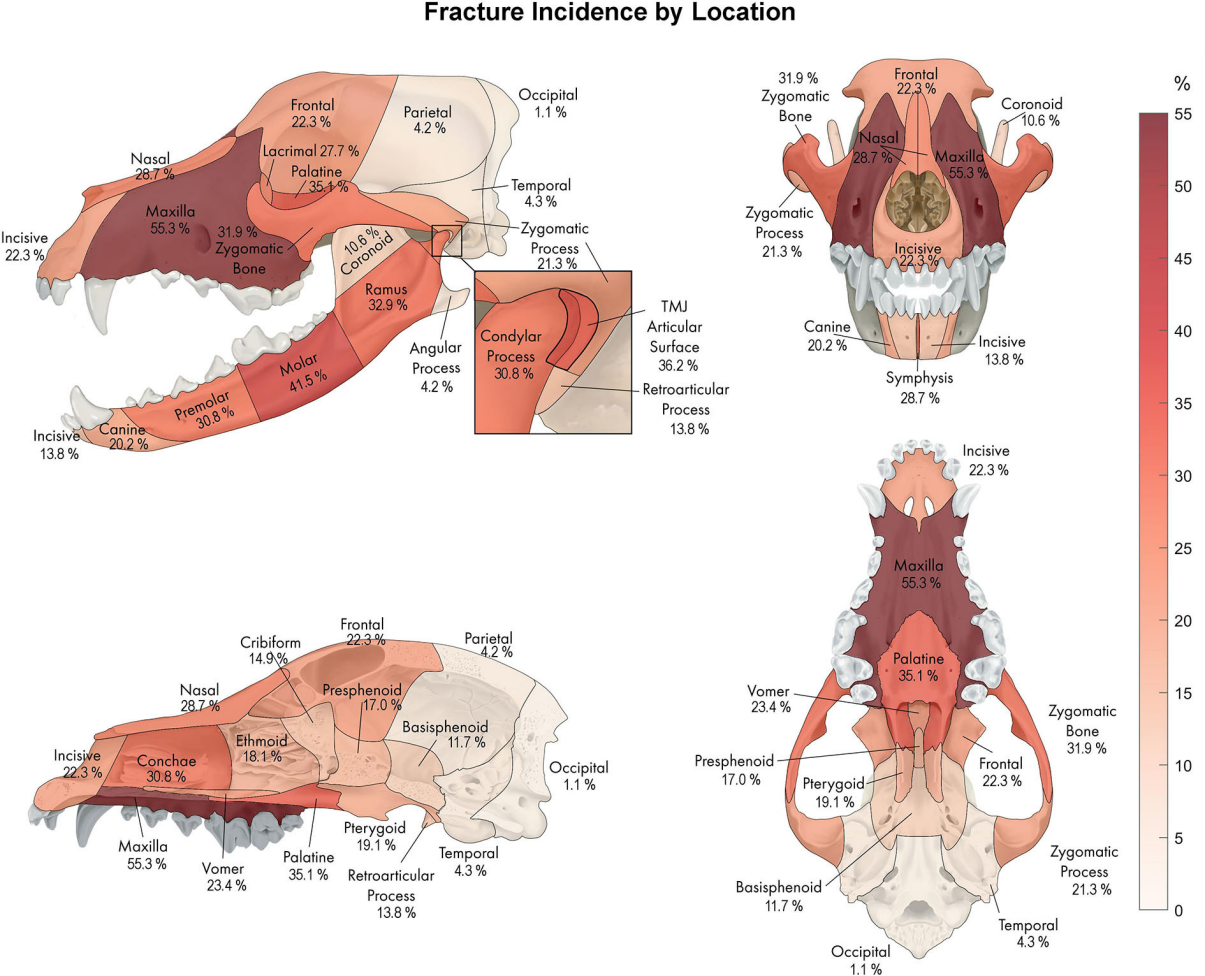
A percentage heat map demonstrating the proportion of CMF fracture locations. Percentages represent the percentage of dogs sustaining a fracture in each location. For example, 55.3% of dogs in this study sustained fractures of the maxillary bone, whereas 1.1% of dogs in this study sustained fractures of the occipital bone.

The most common fracture location was the maxilla (Figure 9), with 55.3% (*n* = 52) of dogs having sustained at least one fracture of this bone. The molar part of the mandible was the second most common fracture region in 41.5% (*n* = 39) of the cases. The least commonly affected location was the occipital bone noted in 1 dog (1.0%). There was no bone or region in the skull that was unaffected in all cases (i.e., no bone/region was fractured in no cases). No attempt was made to determine significance based on possible overlapping of confidence intervals. However, a general trend of increasingly common fractures of the midface (maxilla, zygomatic, palatine, conchae, and nasal bones) as well as the premolar, and molar parts, as well as the ramus and condylar process of the mandible, can be seen in Figure 9. The articular surface of the TMJ was fractured in 36.2% (*n* = 34) of cases.

### Concomitant trauma

**Figure 10:**
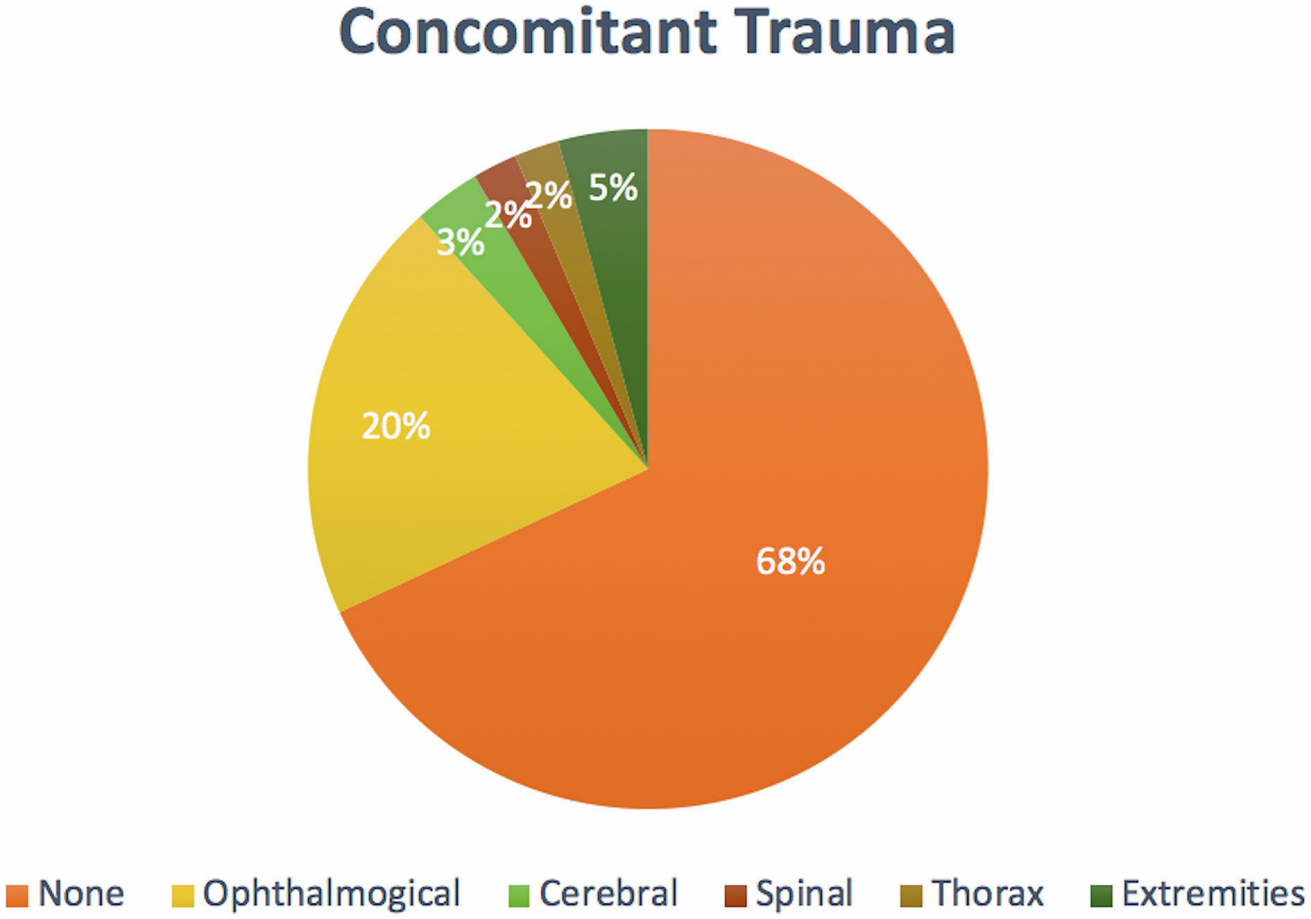
Population distribution of patients sustaining concomitant trauma.

In total, 30 (32.0%) of the dogs sustained concomitant injuries, with the most common associated injuries involving the eye in 19 (20.2%) dogs. Although eye injuries accounted for most of the reported additional injuries, 64 dogs (68.0%) did not sustain concomitant trauma (Figure 10).

### Treatment methods

The most used treatment modality was a conservative method of fracture stabilization or muzzle therapy, accounting for 50 (53.2%) cases (Table 1). Soft tissue closure and selective dental extractions were the second and third most commonly performed interventions accounting for 45 (47.9%) and 26 (27.6%) of dogs, respectively. Bone fragment debridement and interdental wire and composite splint accounted for 19 (20.2%) cases. Salvage procedures were performed in 11 (11.7%) cases. ORIF and circum-mandibular cerclage wire were performed in 6 (6.3%) and 5 (5.3%) dogs, respectively. The least utilized treatment method was a maxillomandibular fixation, which was used in only 2 cases (2.1%). Although a combination of treatment methods was recorded in 28.7% (*n* = 27) of cases, no attempt was made to determine significance based on possible overlapping of confidence intervals.

**Table 1:** Methods of medical intervention.

**Treatment method**	
Muzzle therapy	50 (53.2%)
Soft tissue closure	45 (47.9%)
Dental extractions	26 (27.6%)
Bone fragment debridement	19 (20.2%)
Interdental wire and composite splint	19 (20.2%)
Salvage procedure	11 (11.7%)
ORIF	6 (6.3%)
Circummandibular cerclage wire	5 (5.3%)
MMF	2 (2.1%)

*Note that muzzle therapy accounted for most of the treatment methods*.

### Complications

Out of 94 cases, 81 dogs (86.2%) had at least 1 follow-up examination performed by means of an awake oral examination and/or repeat CT or CBCT scan. The follow-up visits ranged from 1 week to 17 months, following the initial presentation. Follow-up by means of a conventional CT or CBCT scan was performed in 34 cases (42.0%), with an average recheck time of 2.3 months post-trauma. An additional 11 cases had a second follow-up CT or CBCT scan performed, with an average recheck time of 5.1 months.

Complications were recorded in 58 (71.6%) of the follow-up examinations. The most common type of complication was malocclusion, accounting for 32 (55.2%) dogs (Table 2) of which 15.6% were dental malocclusions, and 84.4% skeletal malocclusions or a combination of both. Non-vital teeth and tooth structure defects were reported in 23 (39.7%) and 21 (36.2%) cases. The average amount of complications was 2.4 per dog, with up to 8 reported complications. The least commonly reported complications were bone sequestrum formation and dehiscence, with each being reported in 2 (3.4%) and 4 (6.9%) of the follow-up examinations. Although none of the reported complications had true TMJ ankylosis, 6 (10.3%) dogs developed TMJ anomalies including pseudoankylosis and osteoarthrosis.

**Table 2:** Complications associated with immature craniofacial trauma.

**Complications**	
Malocclusion	32 (55.2%)
Non-vital teeth	23 (39.7)
Tooth structure defect	21 (36.2%)
Delayed healing	11 (19.0%)
Persistent deciduous teeth	10 (17.2%)
Infection	10 (17.2%)
Embedded dentition	9 (15.5%)
TMJ anomalies	6 (10.3%)
Decreased range of motion	5 (8.6%)
Other	5 (8.6%)
Dehiscence	4 (6.9%)
Bone sequestrum	2 (3.4%)

### Fracture healing

Jonckheere-Terpstra trend tests with Spearman correlation analyses revealed significant positive correlations between severity of fragmentation or displacement of the fracture fragments, and worsening fracture healing outcomes (Table 3). If for any given bone or region if the fracture was comminuted, it was less likely to show satisfactory healing on the recheck CT scan. Equally, if a bone or region had minimal or no displacement it had a higher chance of showing evidence of satisfactory healing or being healed in the recheck interim (Table 3). Thirteen bones or specific bone regions did not meet the inclusion criteria, namely the incisive, canine, and premolar region of the mandible, coronoid process, angular process, incisive bone (upper jaw), retroarticular process, occipital bone, temporal bone, parietal bone, vomer, ethmoid, and basisphenoid.

**Table 3:** Spearman correlation between severity of fragmentation and displacement in relation to fracture healing outcomes.

	**Fragmentation**		**Displacement**	
**Bone or region**	***n***	**rho (95%CI)**	***n***	**rho (95%CI)**
Symphysis	10	0.98 (0.95, 1.00)	10	0.98 (0.94, 1.00)
Molar region of mandible	16	0.93 (0.85, 1.00)	16	0.92 (0.84, 1.00)
Ramus	16	0.91 (0.79, 1.00)	16	0.92 (0.82, 1.00)
Condylar process	13	0.87 (0.73, 1.00)	13	0.89 (0.74, 1.00)
TMJ	14	0.94 (0.85, 1.00)	14	0.96 (0.92, 1.00)
Maxilla	22	0.76 (0.55, 0.98)	22	0.74 (0.50, 0.99)
Nasal bone	10	0.98 (0.95, 1.00)	10	0.99 (0.98, 1.00)
Zygomatic bone	11	0.98 (0.96, 1.00)	11	0.98 (0.96, 1.00)
Pterygoid	10	0.97 (0.94, 1.00)	10	0.97 (0.93, 1.00)
Lacrimal bone	12	0.96 (0.96, 1.00)	12	0.95 (0.89, 1.00)
Conchae	14	0.97 (0.93, 1.00)	14	0.95 (0.89, 1.00)
Zygomatic process	10	0.99 (0.96, 1.00)	10	0.99 (0.96, 1.00)
Palatine bone	16	0.95 (0.89, 1.00)	16	0.94 (0.85, 1.00)
Frontal bone	11	0.98 (0.96, 1.00)	11	0.99 (0.96, 1.00)
Presphenoid	10	0.99 (0.97, 1.00)	10	0.98 (0.95, 1.00)
Cribriform plate	10	0.93 (0.82, 1.00)	10	0.92 (0.82, 1.00)

*Note that all of the correlations are within the 95% confidence interval*.

### Fracture location and malocclusion

Out of all the dogs that had follow-up, 32 developed a malocclusion of which 25 had fractured a dentate region of the mandible. No malocclusion was recorded in 28 dogs despite having a fracture of the dentate region of the mandible. Fisher's exact test revealed a significant association between fractures of the teeth-bearing regions of the mandible and malocclusion (*p* = 0.050). No significant associations were observed between upper jaw fractures, non-dentate mandibular region fractures, and malocclusion.

### Intervention and healing outcome

When evaluating the difference in healing outcomes across different treatment modalities, no significant differences between conservative, non-invasive, or invasive repair techniques for any given bone region were found, as determined by the Kruskal-Wallis test. In addition, no reported treatment modality was significantly associated with the development of malocclusion.

### Temporomandibular joint fractures

**Figure 11:**
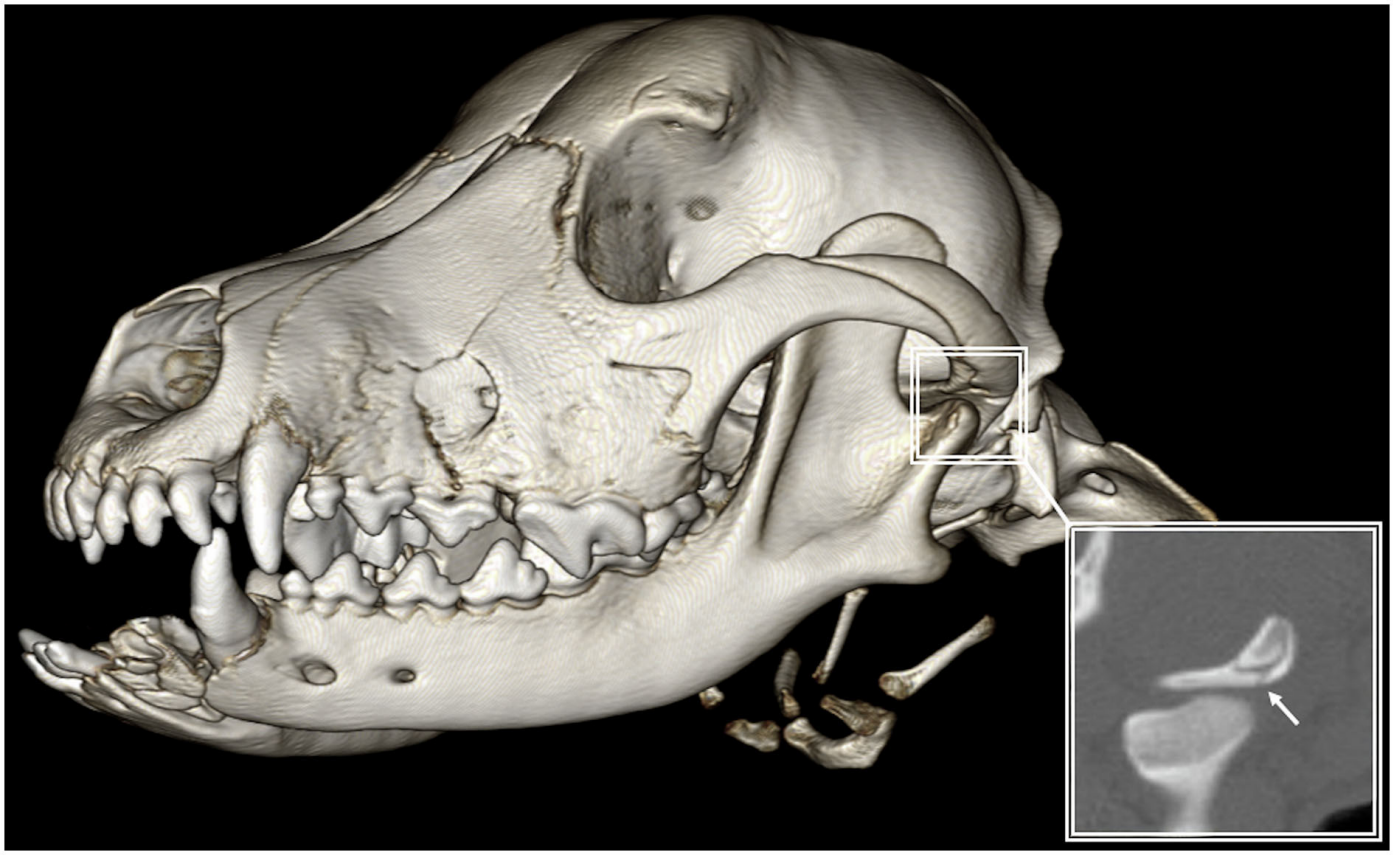
3D-rendered CBCT image of the skull with reconstructed sagittal plane insert of a 6-month-old cross-breed dog. Rostral mandibular trauma was significantly associated with distant fractures of the articular surface (arrow) of the TMJ.

Out of all the dogs that had a fracture involving the TMJ (*n* = 34), 18 dogs had rostral mandibular trauma. A symphyseal joint separation and a fracture of the incisive and/or canine region of the mandible were considered traumatic events for the rostral mandible (Figure 11). In total, 60 dogs had no fracture of the TMJ of which 17 had rostral mandibular trauma. Fisher's exact test revealed a significant association between rostral mandibular trauma and articular surface fractures of the TMJ (*p* = 0.016).

### Additional medical intervention

Out of 81 dogs that had a follow-up examination performed, 45 (55.6%) required additional medical intervention by means of medical management and/or a surgical procedure. For a dog that needed additional intervention, the average number of further treatments was 1.9 per dog. The most commonly performed treatment consisted of dental extractions, performed in 35 (77.7%) of the dogs (Table 4). Antibiotic treatment and debridement were done in 16 (35.5%) and 6 (13.3%) of the cases respectively. Salvage procedures, including partial mandibulectomy and condylectomy, were performed in 4 (8.8%) of the patients that required further treatment. The least commonly performed treatments were odontoplasty, sequesterectomy, and soft tissue closure, each accountable for 2 (4.4%) of the cases. Root canal treatment was completed in 3 (6.6%) cases. Similarly, implant removal or reconstruction with or without the use of rhBMP-2 was performed in 3 (6.6%) of the dogs (Table 4).

**Table 4:** Additional medical intervention.

**Additional treatment**	
Dental extractions	35
Antibiotic treatment	16
Debridement	6
Salvage procedure	4
Root canal treatment	3
Implant removal	3
Reconstruction +/- BMP	3
Odontoplasty	2
Sequesterectomy	2
Soft tissue closure	2

*Note that additional dental extractions were performed in most of the patients that needed additional treatment*.

## Discussion

This retrospective and CT/CBCT-based study examined CMF trauma in immature dogs and included several clinically relevant details on treatment modalities, healing outcomes, complications, and the need for additional treatment in the course of healing. First, the use of CT and/or CBCT were paramount to accurately diagnose the fractured bone or regions in the CMF. Second, fractures in rostral location were significantly associated with fractures at a distant location such as the TMJ. Third, displacement and fragmentation were correlated with healing outcomes, further accentuating the need for CT as an invaluable diagnostic and prognostic tool. In addition, there was no significant difference between conservative, non-invasive, or invasive repair techniques, but complications, although mostly minor, were common and should not be disregarded. Finally, the need for a follow-up examination and imaging in order to address complications was evident.

In agreement with previously performed studies (1, 27), dogs affected by CMF trauma often have multiple bone fractures. This further signifies the importance of CT for complete and accurate diagnosis which is considered the gold standard in people and veterinary species (20, 22, 23, 33). The use of plain skull radiography typically underdiagnoses the presence of fractures in maxillofacial trauma, as was demonstrated previously in dogs (20) and humans (33). This has immediate and important implications for the treatment plan and prognosis. This study revealed, for example, that rostral mandibular trauma is significantly associated with articular surface fractures of the TMJ. Noticeable is the fact that not a single patient developed true TMJ ankylosis despite 36.2% of cases having articular surface fractures of the TMJ. These findings are in agreement with our previous report (34) and the reported post-traumatic TMJ ankylosis in people that is rare, with an annual incidence rate of ~0.4% (35).

The maxilla was the most commonly fractured bone with an incidence of 55.3%, which is similar to previous reports (53.3%) (1). As described elsewhere (8, 36), the maxilla of the dog is a prominent and exposed structure and is, therefore, more susceptible to traumatic insults than other CMF structures. The premolar/molar part of the mandible is similarly exposed to traumatic insults, especially those occurring from the side or the bottom direction. The mandible was most often fractured in the molar region (41.5%), followed by the ramus (32.9%) and premolar region (30.8%) of the mandible. This is in agreement with previously conducted research where the molar region was fractured in 41.2–47.1% of the fractures (1–3). In another study, the canine region (46.4%) was the most commonly affected region in mandibular fractures (6) in juvenile dogs. Tooth morphology may influence the location of the mandibular fracture (37, 38). Small dogs have proportionally larger mandibular first molar teeth relative to mandibular height compared to larger dogs (39). Fractures typically propagate along the path of least resistance, but the direction and extent of this path is a complex relationship between environmental and intrinsic factors (40). Given that the bone height of the mandible is short near the mandibular first molar tooth (39), it is expected for a fracture to initiate or propagate along this area. Taken together, the more exposed bones of the CMF region, such as the maxillae and the premolar-molar regions of the mandibles tended to sustain the most fractures following trauma.

Increase in severity of fragmentation and displacement were associated with an unsatisfactory healing outcome. The importance of appropriate treatment of severely displaced and fragmented fractures should not be underappreciated despite the fact that young dogs have excellent bone healing capacity (41). Muzzle coaptation may not be the best course of action in these patients, as it provides relatively weak support to the fracture fragments. Open reduction and internal fixation for severely displaced and fragmented fractures may be considered. The introduction of titanium plate fixation fundamentally changed the treatment algorithm for maxillofacial trauma and improved surgical outcomes (42). The qualities of titanium that make it ideal for internal fixation include its intrinsic mechanical strength, pliability, and biocompatibility (43). The long-term effects of rigid internal fixation in the growing, pediatric facial skeleton are unclear and remain a controversial subject, as some fear permanent fixation alters craniofacial growth and some studies report a higher complication rate with ORIF vs. a closed approach (44–48). The human medical literature reports that the intrinsic qualities of pediatric facial fractures (e.g., accelerated healing rate, skeletal growth, and bone remodeling potential) make rigid fixation with biodegradable material possible (49). In addition, the applicability of resorbable hardware in the pediatric trauma and craniofacial surgery fields has increased owing to advances in biodegradable plating technology. Taken together, in our study, utilization of ORIF in immature dogs appears to be limited to exceptional cases that exhibit severe fragmentation and displacement of fractured bones where the placement of plates and screws is feasible without the interference of developing or currently present dental structures, but with an overall good outcome.

We demonstrated that neither treatment modality was significantly associated with favorable or unfavorable healing outcomes or an increasing trend toward the development of malocclusion. Although no statistically significant association between treatment modality and outcome was found, it does not imply that either method can be used in every age group and the outcome is expected to be the same, e.g., a 2-month-old dog may not be a good candidate for ORIF or interdental wire and splint. In addition, previous research demonstrated an overall good healing outcome for juvenile dogs that sustained mandibular fractures where muzzling as a non-invasive treatment method was used in 72.4% of dogs with a relatively rapid mean healing time of 21 ± 9 days (6). There was, however, a significant correlation between dentate mandibular fractures and the development of malocclusion. Malocclusion was the most reported type of complication (55.2%), consistent but higher than previously reported (34.0–37.9%) (4, 6). This may be due to the combining of dental and skeletal malocclusions in data collection. The need for additional medical intervention and the highest reported treatment modality being additional selective dental extractions is further explained by the relatively high proportion of non-vital teeth, tooth structure defects, persistent deciduous, embedded dentition, and malocclusions, all of which may incur the necessity for dental extractions. This is in agreement with a previous study that reported that a relative high proportion of juvenile dogs with mandibular fractures developed dental anomalies of developing tooth buds in or near the fracture line (73.5%) and 10 out of 11 dogs required additional dental extractions (7). Complications were common (71.6%) but overall considered minor, and manageable with conservative treatment or additional medical intervention in 55.6% of the cases. This highlights the importance of monitoring the development of dental abnormalities and/or skeletal malocclusion until permanent teeth have erupted and jaw growth is completed in dogs that sustained CMF trauma.

The limitation of this study is inherent to its retrospective design as well as the fact that not all dogs had a follow-up examination performed or repeat imaging. In addition, the cases included in this study were assessed at a tertiary referral institution, which could have affected the types of CMF trauma included. For example, very mild cases may not have been referred to our institution if the primary veterinarian felt capable of treating the case. Conversely, very severe cases may have died or been euthanized prior to referral.

In conclusion, immature CMF trauma presents unique challenges in diagnosis and management. The developing CMF bones and associated dental development should be considered when interpreting imaging and formulating treatment plans as well as evaluating prognosis. In the vast majority of cases, a non-invasive approach was used with an overall good healing outcome, albeit with relatively high, but mostly minor, healing complications. Minimally invasive and invasive treatment methods were reserved for selected cases that exhibited moderate to severe displacement and resulted in an overall good outcome. Finally, it is important to note that the data presented here suggest that when managing immature CMF trauma, additional treatment, even if minor, may be warranted. Thus, the need for a comprehensive assessment of the entire CMF region during the initial visit as well as the follow-up, preferably using CT, to determine the appropriate treatment is underscored.

## Data availability statement

The raw data supporting the conclusions of this article will be made available by the authors, without undue reservation.

## Ethics statement

Ethical review and approval was not required for the animal study because the study is retrospective in nature and included clinical cases, hence, it is exempt from IACUC requirements. Written informed consent for participation was not obtained from the owners because the study is retrospective in nature and, hence, it is exempt from written informed consent.

## Author contributions

EW: study concept and design, image analysis, data acquisition, analysis, and interpretation. JG: image analysis, data acquisition, analysis, and interpretation. BA and FV: study concept, design, and data interpretation. PK: data analysis and interpretation. All authors contributed to the article, drafting of the manuscript, and approved the submitted version.

## Conflict of interest

The authors declare that the research was conducted in the absence of any commercial or financial relationships that could be construed as a potential conflict of interest.

## Publisher's note

All claims expressed in this article are solely those of the authors and do not necessarily represent those of their affiliated organizations, or those of the publisher, the editors and the reviewers. Any product that may be evaluated in this article, or claim that may be made by its manufacturer, is not guaranteed or endorsed by the publisher.
